# Mid-term results of ponseti method for the treatment of congenital idiopathic clubfoot - (A study of 67 clubfeet with mean five year follow-up)

**DOI:** 10.1186/1749-799X-6-3

**Published:** 2011-01-12

**Authors:** Milind M Porecha, Dipak S Parmar, Hiral R Chavda

**Affiliations:** 1 Orthopedic Department, M.P.Shah Medical College, Guru Govind Singh Hospital, Jamnagar - 361008. Gujarat. India; 2 Department of orthopedics, M.P.Shah Medical College, Guru Govind Singh Hospital, Jamnagar - 361008. Gujarat. India; 3 Department of anesthesiology, M.P.Shah Medical College, Guru Govind Singh Hospital, Jamnagar - 361008. Gujarat. India

## Abstract

**Background:**

Long-term success reports by Dr. Ponseti with the Ponseti method in the treatment of congenital idiopathic clubfoot have led to a renewed interest in this method among pediatric orthopedists. The purpose of this study is to evaluate mid-term effectiveness of Ponseti method for the treatment of congenital idiopathic clubfoot.

**Material and Methods:**

A total of 49 patients (67 clubfeet) were treated by Ponseti method by single orthopedic surgeon during the period of October 03 to July 07 and were studied prospectively up to July 10 (mean follow up period 5 years, minimum follow-up period of 3 years). Age at the initiation of the treatment, gender, bilaterality, severity of the initial clubfoot deformity measured by Pirani Severity Score System, total numbers of Ponseti casts before the tenotomy, details of tenotomy, compliance with brace and CTEV shoes were examined. Passive range of movements and look of club foot are evaluated with mean 5 years follow-up.

**Results:**

We followed the functional Ponseti Scoring System and got good to excellent results in 44 patients - 89.79% (58 clubfeet - 86.56%) at mean five year of follow up. Parents of 32 patients (65.30%) accept the look of the clubfoot nearly normal and parents of 12 patients (24.49%) accept the look of clubfoot as normal. Of the 49 patients who responded to initial Ponseti casting, 14 patients - 28.57% (19 clubfeet - 28.35%) had relapse at varying age; out of which 9 patients - 64.29% (10 clubfeet - 52.63%) were corrected by Ponseti casting method, while 5 patients - 35.71% (9 clubfeet - 47.37%) were resistant to Ponseti method. Poor compliance with the Denis Browne splint was thought to be the main cause of failure in these patients.

**Conclusion:**

Ponseti method is a safe and satisfactory treatment for congenital idiopathic clubfoot with mid- term effectiveness.

## Background

Congenital idiopathic clubfoot is a complex deformity that is difficult to correct. The deformity has four components: Ankle Equinus, Hindfoot Varus, Forefoot Adductus, and Midfoot Cavus. The goal of the treatment is to reduce or eliminate all the components of clubfoot to obtain painless, plantigrade, pliable and cosmetically and functionally acceptable foot within the minimum time duration with least interruption of the socio-economical life of the parent and child.

There is nearly universal agreement that the initial treatment of the clubfoot should be non-operative regardless of the severity of the deformity. Historically, the treatment consists of forcible serial manipulation and casting with pressure applied over the calcaneo-cuboid joint as describe by the Kite [1]. If the deformity did not respond then most of the surgeons go through Postero-Medial Release of the soft tissue. Although all of these methods have the potential to be successful when applied correctly, most of the authors have reported a long-term success rate of only 15% to 50% [2,3]. A notable exception is the Ponseti method [4] which includes serial corrective manipulation, a specific technique of the cast application, and a possible percutaneus Achilles tenotomy. The method has been reported to have short-term success rate approaching 90% and mid to long-term results are also equally impressive [4,5]. Cooper and Dietz, in a review of the cases of forty-five patients who had been treated by Ponseti and followed for a mean of thirty years, found that, with the use of pain and functional limitation as the outcome criteria, thirty-five patients (78%) had achieved an excellent or good outcome [5].

The unsatisfactory results associated with complete soft tissue release at 10 to 15 years of follow-up [6-8] and the long-term success reported with the Ponseti method have led to a renewed interest in this method among pediatric orthopedists. Despite this interest, long-term success with the Ponseti method when it has been used by other orthopedists has not been demonstrated till recently in world literature.

The purpose of this study was to evaluate the mid-term effectiveness of the Ponseti method [4] for the treatment of congenital idiopathic clubfoot.

## Materials and methods

A total of 49 patients (67 clubfeet) were treated by Ponseti method by single orthopedic surgeon during the period of October 03 to July 07 and were studied prospectively up to July 10 (mean follow up period 5 years, minimum follow-up period of 3 years) at our institute after taking informed consent of parents of patients prior being included into the study and was authorized by the local ethical committee. The study was performed in accordance with the Ethical standards of the 1964 Declaration of Helsinki as revised in 2000. Clubfoot associated with myelocele, meningomyelocele, arthrogryposis multiplex congenital and other neuromuscular causes were excluded, to avoid the effect of neuromuscular imbalance on treatment results. Age at the beginning of the treatment, gender, pattern of involvement of the foot, severity of the foot deformity according to Pirani Severity Score [9], total number of the casts applied before tenotomy, details of tenotomy, details of Denis-Browne Splint and CTEV shoes were noted.

Clinical assessments included: the incidence of residual and recurrent deformities, passive range of movement (measured by goniometer), appearance, muscle power, calf atrophy, foot size and other complications. Functional assessments included: gait, functional limitation, shoe wear, pain and patient satisfaction. We do not include radiological assessment in our study. The Ponseti scoring system [4] for functional results was used, with 100 points indicating a normal foot. This includes a maximum score of 30 points for amount of pain; of 20 points each for level of activity and patient satisfaction; and of 10 points each for motion of the ankle and foot, position of the heel during stance, and gait. For Satisfaction and Function category, data has been recorded from the patients' parents considering patient as minor. (Table 1)

**Table 1:** Functional Scoring System According to Dr. Ponseti [4]

Category	Points
**Satisfaction (20 points)**	
I am ...	
1. very satisfied with end results	20
2. satisfied with end results	16
3. neither satisfied nor unsatisfied with end results	12
4. unsatisfied with end results	08
5. very unsatisfied with end results	04

**Function (20 Points)**	
In my daily living my club foot...	
1. Does not limit my activities	20
2. Occasionally limit my strenuous activities	16
3. Usually limits me in strenuous activities	12
4. Limits me occasionally in routine activities	08
5. Limits me in walking	04

**Pain (30 points)**	
My club foot...	
1. Is never painful	30
2. Occasionally causes mild pain during strenuous activities	24
3. Usually is painful after strenuous activities only	18
4. Is occasionally painful during routine activities	12
5. Is painful during walking	06

**Position of heel when standing (10 points)**	
1. Heel varus 0 degree or some heel valgus	10
2. Heel varus 1-5 degree	5
3. Heel varus 6-10 degree	3
4. Heel varus >10 degree	0

**Passive motion (10 Points)**	
1. Dorsiflexion	1 point per 5 degree (up to 5 points)
2. Total varus-valgus motion of heel	1 point per 10 degree (up to 3 points)
3. Total inversion-eversion of foot	1 point per 50 degree (up to 2 points

**Gait (10 Points)**	
1. Normal	6
2. Can toe walk	2
3. Can heel walk	2
4. Limp	-2
5. No heel strike	-2
6. Abnormal toe off	-2

The results were graded as Excellent (90-100 points), Good (80-89 points), Fair (70-79 points) and Poor (less than 70 points) [4]. Poor and fair results were considered failures and needed further management for residual or recurrent deformity.

## Treatment regimen

The Ponseti method is used at our institution according to following regimen. Treatment is started as soon as the skin condition permits and consists of gentle manipulation of the foot and the serial application of long leg plaster casts at weekly interval without the use of anesthesia, as described by Ponseti [4].

In all patients, the cavus is corrected first by supinating the forefoot and dorsiflexing the first metatarsal. Failure to supinate the forefoot as the first step ultimately leads to incomplete correction of the clubfoot. To correct the varus and adduction, the foot in supination is abducted while counter-pressure is applied with the thumb against the head of the talus. Four to eight long leg casts, changed weekly after proper manipulation of the foot, are usually sufficient to obtain good correction. In the last cast, the foot should be markedly abducted up to 70 degree without Pronation. This position is crucial in obtaining complete correction and in helping to prevent early recurrence.

If residual equinus is observed after the adduction of the foot and the varus deformity of the heel has been corrected, a simple percutaneus tenotomy of the Achilles tendon is performed. We prefer to perform the tenotomy in the operating room with the patient under general anesthesia, which allows optimal analgesia for the infant. This setting also provides the surgeon with the controlled environment; with hopefully optimize the safety of this procedure. This approach differs from the Ponseti [4] who prefers that the Achilles tenotomy should be done in the clinic with topical and/or local anesthesia. Tenotomy is performed when 15 degree of the dorsiflexion is not obtained with the use of casts after correction of varus and adductus deformities. After the tenotomy, an additional long leg cast with knee flexed in 90 degree is applied and left in place for three weeks to allow for healing of the tendon.

**Figure 1:**
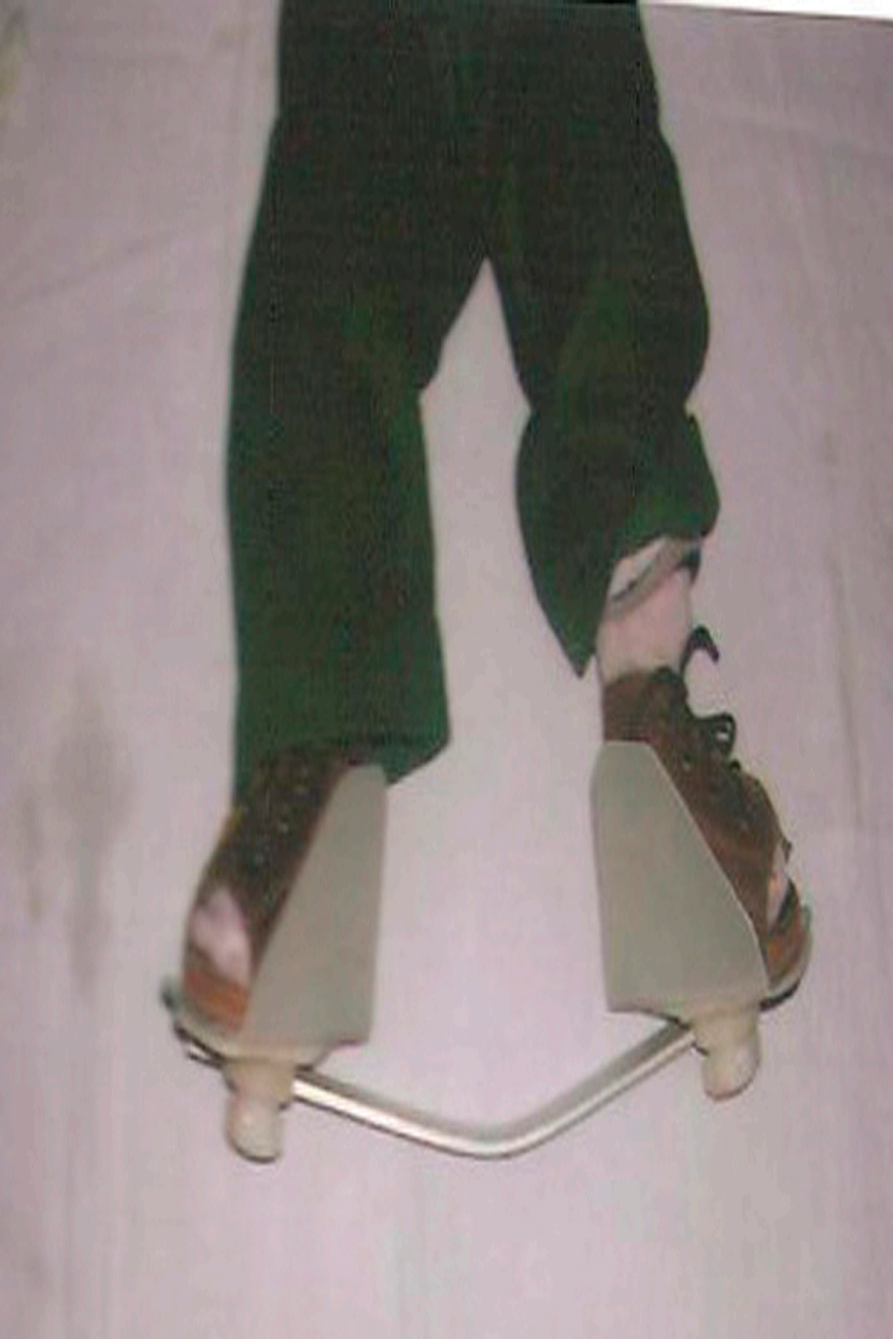
**Denis- Browne Splint for bilateral clubfoot**.

A Denis-Browne splint is used to prevent relapse of the deformity. This is best accomplished with the feet in well-fitted, open-toed, medial bar, high-top straight-last shoes attached to Denis-Browne bar of approximately the length between the child's shoulders. (Figure 1) The splint maintains the corrected foot in 70 degree of external rotation to prevent recurrence of the varus deformity of the heel, adduction of the foot, and toeing in [4]. The ankle should be in dorsiflexion in an attempt to prevent equinus; and this is accomplished by bending the bar 10-15 degrees with the convexity of the bar distally directed. If the deformity is unilateral, the normal foot is placed in 45 degree of external rotation. The knees were left free to stretch the gastrocnemius and to provide a corrective force to the other foot.

The splint were retained until the walking age for twenty three hours a day, and thereafter worn only at night until the age of 5 years. By day, shoes with an open toe box, straight medial border, lateral flaring of the sole and reverse Thomas heels were used until the age of 5 years. This approach differs from that of the Ponseti [4] who prefer to apply the Denis-Browne splint 23 hours a day for three months and then at night (12-14 hours) for three years. Non-compliance was defined as the inability to adhere to the above mentioned criteria and also delay in changing the splint and shoes as the foot size changed.

The parents were instructed to perform range of motion exercises for the ankle and foot when it was out of the brace. Two exercises were taught to the parents. In the first exercise the infant was made to squat on level ground while being supported by the parents. This brought the ankle in dorsiflexion and prevents equinus deformity. In the second exercise the parent uses one hand to stabilize the leg with knee bent. The other hand is used to grasp the foot and then place the ankle into maximum dorsiflexion followed by planter flexion.

The exercises were performed twice a day till the weight bearing age (when the brace was applied for twenty three hours a day) and five times daily for the next three years (when the brace was applied for twelve hours at night). The parent repeats this exercise twenty times at a seating.

The patients were followed up on a weekly basis during the initial stages of treatment. After orthosis was applied, the patient was seen on a monthly basis for three months and then once every three months till the patients was three years of age. The patient was also followed up every six moths to one year till 5 years and then after 1-2 years till skeletal maturity is achieved.

## Results

A total number of 49 patients with 67 clubfeet were treated and followed for mean of five years. Out of 49 patients, 39 patients (79.59%) were male, thus male-female ratio is 3.9. Out of 49 patients, 18 patients (36.73%) had bilateral involvement while 31 patients (63.27%) had unilateral involvement out of which 17 (54.84%) had right foot involvement and 14 (45.16%) had left foot involvement. No relationship had been found with birth order or family history.

While beginning of the treatment, 42 patients (85.71%) are in between 0-12 weeks of the age (mean 2 weeks), 5 patients (10.20%) are in between 13-24 weeks of age (mean 15 weeks) while 2 (4.08%) patient are in between 25-36 weeks of age (mean 34 weeks). At the commencement of treatment, of the 18 bilateral clubfeet patients (36 clubfeet) 17 children (34 clubfeet) had Pirani severity score of six, and one children (2 clubfeet) had a Pirani score of five. In unilateral group the mean Pirani score was 5.83 (range 5-6).

The mean Mid Foot Score and Hind Foot Score for the entire group was 2.8 (range 2.5-3) and 2.76 (range 2-3) respectively. The mean number of the casts that were applied to obtain correction was 6.8 (range 6-8). The more severe the initial deformity and the treatment initiation after 12 weeks of the age, the more casts were required to obtain correction. 47 children (95.91%) needed percutaneus tenotomy, 18 in the bilateral group and 29 in the unilateral group. The mean Mid Foot Score and Hind Foot Score for the entire group at the time of tenotomy was 0.5 and 2.5 respectively. There was no delay between final cast removal and fitting of D-B splint. The mean duration of the treatment up to application of the D-B Splint was 9.6 weeks. Initial correction was obtained in all 67 clubfeet (100%) with the Ponseti method.

Fourteen children - 28.57% (19 feet - 28.35%) had a relapse of the deformity. Patient age at the time of relapse, bilateralism or unilateralism of the relapse foot, relapse foot deformity, treatment offered to relapsed foot, immediate results of the offered treatment accessed by Pirani Severity Score, and results at mean 5 year follow-up accessed by Ponseti Functional Scoring System were given. (Table 2)

**Table 2:** Details of the Relapse Foot

Patient's age at relapse (In months)	Bilateralism/Unilateralism at the initiation of the treatment	Side of relapsed foot	Relapse deformity	Treatment offer to correct the deformity	Result of the Treatment	Result at five year of follow up
9	1. Bilateral	Left	Adductus & Varus	4 Ponseti casts	Good	Good
	
	2. Bilateral	Left	Adductus & varus	3 Ponseti casts	Excellent	Good
	
	3. Unilateral	Right	Adductus	2 Ponseti casts	Excellent	Excellent

12	1. Bilateral	Left	Adductus & Varus	3 Ponseti casts	Excellent	Excellent
	
	2. Unilateral	Left	Adductus	2 Ponseti casts	Excellent	Excellent

	3. Unilateral	Right	Adductus & Varus	3 Ponseti casts	Excellent	Good

18	1. Bilateral	Left	Equinus	Repeat tenotomy & 3 week cast	Excellent	Excellent
	
	2. Bilateral	Both	Adductus & Varus	4 Ponseti casts	Excellent	Good

24	1. Bilateral	Both	All four deformities	8 Ponseti casts	Poor	Poor

30	2. Unilateral	Right	Adductus & Varus	3 Ponseti casts	Excellent	Good
	
	3. Bilateral	Both	All four deformities	10 Ponseti casts	Poor	Poor

36	1. Bilateral	Both	All four deformities	8 Ponseti casts	Poor	Poor
	
	2. Bilateral	Both	All four deformities	8 Ponseti casts	Poor	Poor
	
	3. Unilateral	Left	All four deformities	8 Ponseti casts	Poor	Poor

The original correction was recovered with the use of repeat application of serial casts in 8 patients (9 clubfeet) while 5 patients (9 clubfeet) were resistant to Ponseti serial cast manipulation and were offered surgery in the form of Postero-medial release; but parents of the patients were not willing for the surgery and thus had poor functional outcome at mean five year of follow-up. All the 8 patients (9 clubfeet) who respond well to repeat application of serial casts were from the 0-12 weeks of the age group while beginning of the treatment. Out of 5 patients resistant to Ponseti serial cast manipulation 3 were from the 13-24 weeks of the age group while beginning of the treatment, while 2 was from the 25-36 weeks of the age at the initiation of the treatment. Thus, relapse is more severe when occurred and not respond to traditional Ponseti casting method in the patients whom treatment initiation was done after 12 weeks of the age.

One patient (left clubfeet) developed relapse in the form of equinus deformity at the age of 18 months for which repeat percutaneus tenotomy was done and above knee cast was applied with 15 degree dorsiflexion of ankle, 60 degree of abduction of foot and 90 degree knee bent for 3 weeks. Patient had excellent functional outcome at final follow-up.

Thus, of 14 relapsed patients, 9 patients - 64.29% (10 clubfeet - 52.63%) had excellent to good functional outcome and 5 patients - 35.71% (9 clubfeet - 47.37%) had poor functional outcome according to Ponseti Functional Scoring System [4] at the mean five year follow-up. The splint compliance was compromised in all the relapsed cases. In 9 patients the Denis - Browne splint was used infrequently and it was never used in 5 patients.

**Figure 2:**
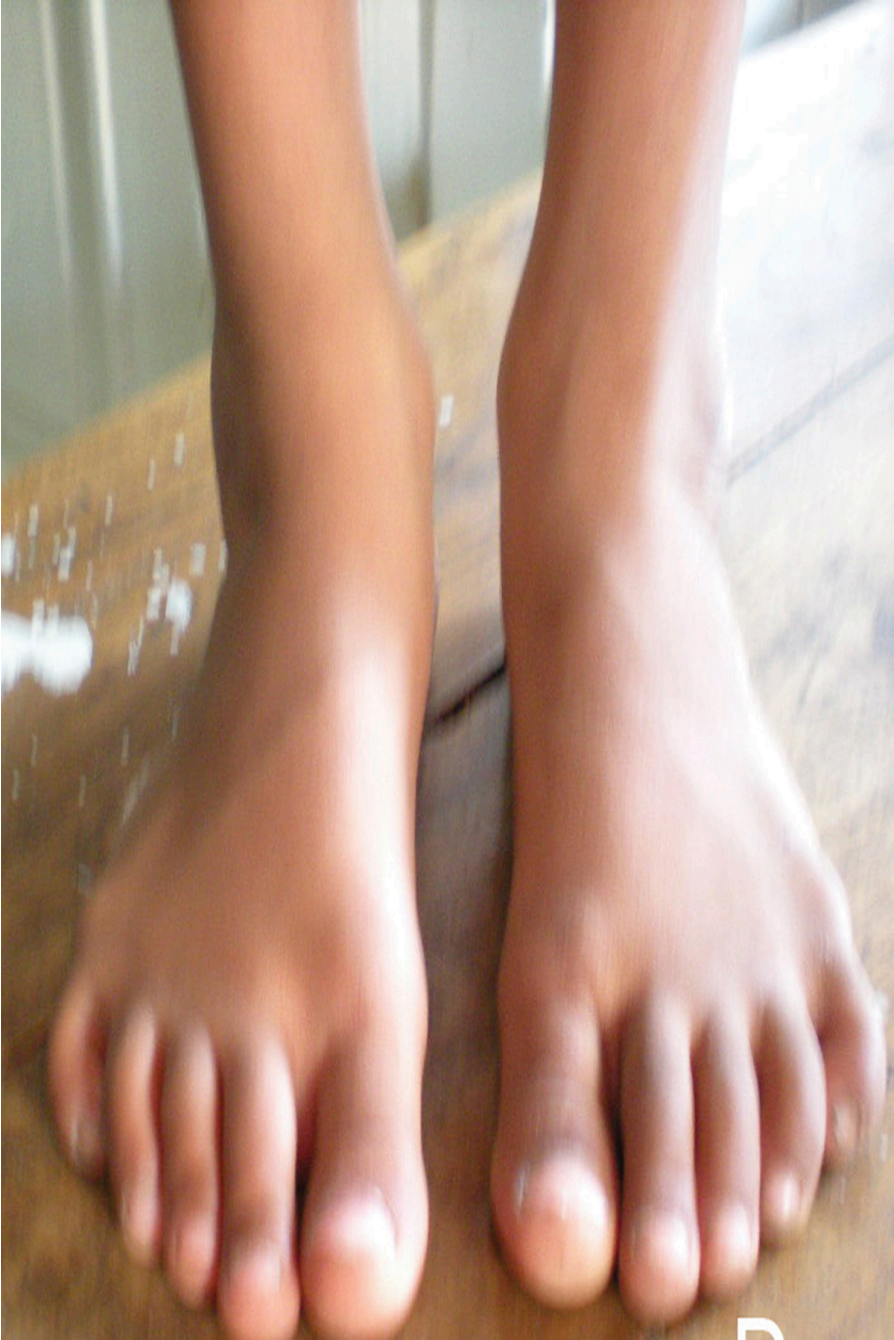
**Front look of bilateral clubfoot at 5 year follow-up**.

**Figure 3:**
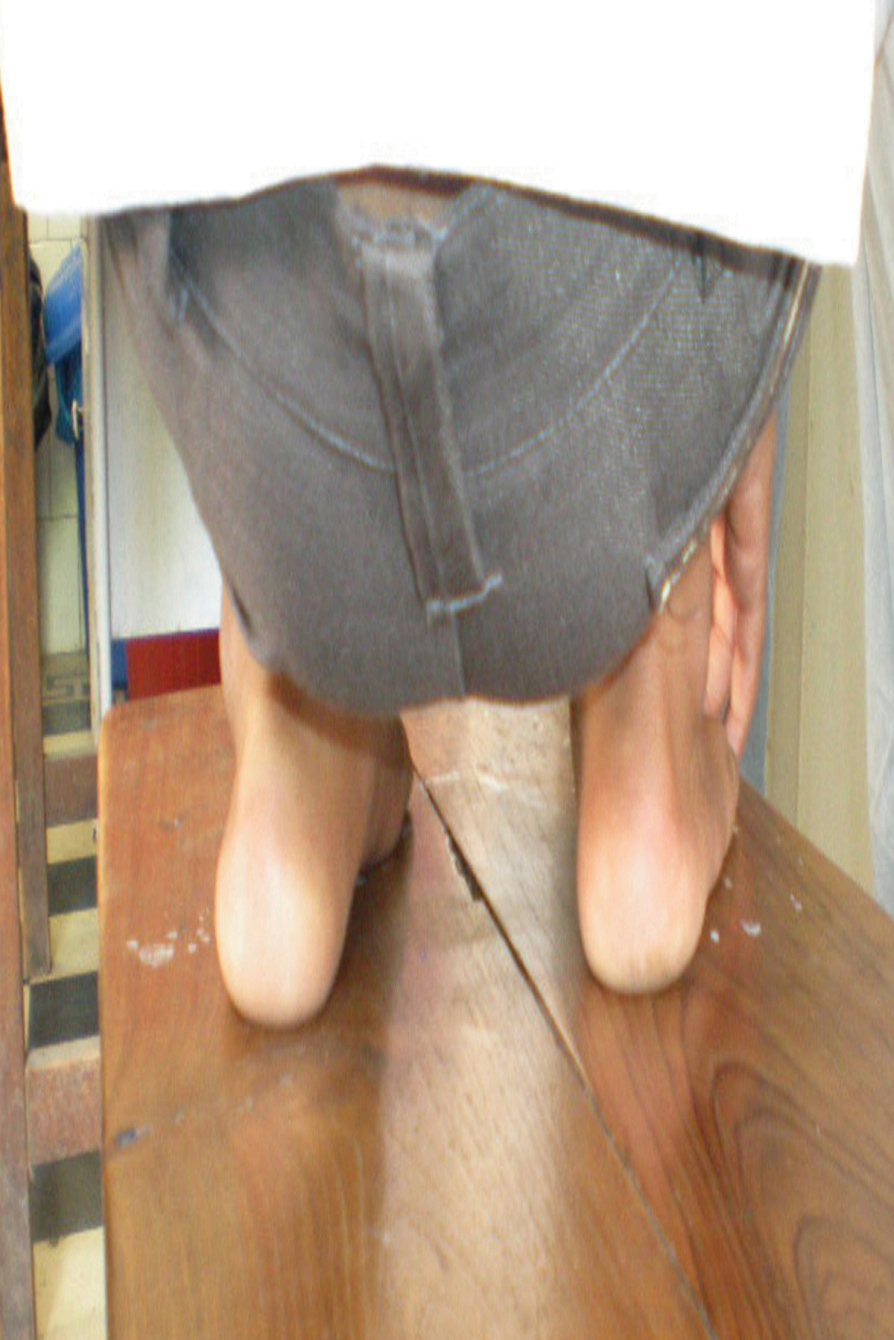
**Back look of bilateral clubfoot at 5 year follow-up**.

At the mean of five year follow-up, we found nearly normal passive range of motion in 44 patients - 89.79% (58 clubfeet - 86.56%). Parents of 32 patients (65.30%) accept the look of the clubfoot nearly normal and parents of 12 patients (24.49%) accept the look of clubfoot as normal. We followed the functional Ponseti Scoring System [4] and got good to excellent results in 44 patients (89.29%) at mean five year of follow up. (Figure 2 & Figure 3)

Few complications were encountered. Two children had a plaster sore on the lateral aspect of the skin overlying the talar head. This healed with local dressing only. The mean time to heal the sore was 7 days (range 6-8 days). The corrective manipulation and cast was not applied till the sore heal. However, we don't encounter any allergic reaction to the soft roll, any transitory discoloration of the toes following tenotomy and correction of equinus, serious bleeding following tenotomy or any wound problems with percutaneus incision.

## Discussion

In 1948, Ponseti proposed reducing the idiopathic clubfoot deformity with successive manipulation and casts. Although treatment with cast is a very old method for clubfoot, Ponseti's method is based on strict rules established from anatomic evidence.

The major concern with the operative treatment of congenital clubfoot is functional outcome. Extensive open surgery like postero-medial release is commonly associated with long-term stiffness and weakness which is avoided by the Ponseti technique [6-8]. Aronson and Puskarich studied the disability associated with various clubfoot treatment options. Their results showed that patients who underwent casting only and patients who had additional percutaneus heel cord lengthening had the least deformity and disability [7].

The Ponseti treatment of clubfoot has three phases: the corrective phase involves application of casts, the maintenance phase where splint fitting is emphasized and the transition phase where the splints are discontinued and regular foot wear allowed. Problems can occur in any phase due to many causes: incorrect casting technique, improper tenotomy, under-corrected deformity, ill-fitting splints, lack of understanding and poor compliance of patients' parents due to poor socio-economy can all affect a successful outcome.

The relapse rate in fourteen cases in our study shows the initial learning curve with this technique. There were more relapse on the left side and this may reflect right hand dominance of the treating surgeon. Thus, a more abduction force may be required to correct the left foot when the left hand is the abduction side.

There are three main issues which lead to inferior results with this technique: splint compliance, splint fitting and under correction of the ankle equinus.

Poor splint compliance was a major issue especially in children coming from low socio-economic strata and where the parents education level was poor. Out of 14 relapses, in 9 patients Denis-Browne splint was used infrequently and it was never used in 5 patients. We feel that although the foot morphology improves with rigid adherence to the casting technique it is the post-correction phase which needs careful attention and close follow up to ensure a successful outcome. We tried to nullify poor splint fitting by providing D-B splint of correct size from a single manufacturer directly under our observation. We now advocate tenotomy in every case to achieve at least 15 degrees of ankle dorsiflexion. This is a critical step as frequently equinus is the first sing of recurrence.

Although 92-98% successful short-term results has been reported for the treatment of idiopathic clubfoot [8,10,11] with Ponseti method, documentation of the long term results of the technique when it has been used by other orthopedists are fewer [4,5]. We tried to evaluate mid-term results for congenital idiopathic clubfoot treated by Ponseti method and are satisfied with the outcome at mean five year of follow-up.

## Competing interests

The authors declare that they have no competing interests.

## Authors' contributions

MP is the single orthopedics surgeon who performs the casting technique in all the patients. DP participate and analysis the study. HC designed and coordinated and drafted the manuscript. All authors read and approved the final manuscript.
